# Prolonged cholestasis following endoscopic retrograde cholangiopancreatography, a rare complication of contrast agent induced liver injury: A case report and literature review

**DOI:** 10.1097/MD.0000000000018855

**Published:** 2020-01-17

**Authors:** Cheng-Kuan Lin, Wen-Chih Huang

**Affiliations:** a Division of Gastroenterology and Hepatology, Department of Internal Medicine; b Department of Pathology, Far Eastern Memorial Hospital, New Taipei City, Taiwan.

**Keywords:** cholestasis, cholestyramine, contrast agent, endoscopic retrograde cholangiopancreatography, ursodeoxycholic acid

## Abstract

**Rationale:**

Prolonged cholestasis is a rare complication associated with endoscopic retrograde cholangiopancreatography (ERCP).

**Patient concerns:**

A 68-year-old man who presented with worsening cholestasis after ERCP for the removal of a common bile duct stone.

**Diagnosis:**

Total bilirubin increased up to 35.2 mg/dL after the 21st day post-ERCP. A percutaneous liver biopsy was performed and drug-related cholestasis was suspected as occurring as a result of the contrast agent.

**Interventions:**

Oral ursodeoxycholic acid and cholestyramine were prescribed to the patient.

**Outcomes:**

By the 7th week post-ERCP, the patient's symptoms and markers of physiological health began to resolve. The bilirubin returned to normal levels on the 106th day post-ERCP. We reviewed the literature for studies of 9 patients with jaundice more than 30 days post-ERCP, the peak of total serum bilirubin occurred on 16th ± 7th days and the recovery followed after mean time of 54th ± 22th days.

**Lessons:**

Although the cholestasis was prolonged, the outcome was favorable after medical therapy. There were no long-term consequences for the patient.

## 1 Introduction

Endoscopic retrograde cholangiopancreatography (ERCP) is an invasive technique used to evaluate and treat biliary and pancreatic diseases.^[1]^ Potential complications associated with ERCP include pancreatitis, hemorrhage, cholangitis, cholecystitis, perforation, and cardiopulmonary difficulties. Miscellaneous rare complications include ileus, hepatic abscess, pneumothorax, pneumomediastinum, duodenal hematoma, portal venous air embolism, impaction of therapeutic devices, and stent-related issues.^[2]^ In this report, we discuss a rare case of prolonged cholestasis associated with severe pruritus following ERCP after the removal of a common bile duct stone.

## 2 Case presentation

A 68-year-old Chinese male worker presented with intermittent epigastralgia and tea-color urine for 1 week. He denied any past and family history of hepatitis, concomitant drug use or alcohol consumption. Physical examination revealed icteric sclera and epigastric tenderness. Laboratory findings were white blood cell count of 6100/μL, aspartate aminotransferase level of 76 IU/L (normal value, <37), alanine aminotransferase level of 226 IU/L (normal value, <41), total bilirubin level of 10.2 mg/dL (normal value, <1.5), alkaline phosphatase level of 179 IU/L (normal value, <129), and γ-glutamyl transpeptidase level of 186 IU/L (normal value, <61). Abdominal sonography revealed gallbladder stones with a dilated biliary tree.

**Figure 1:**
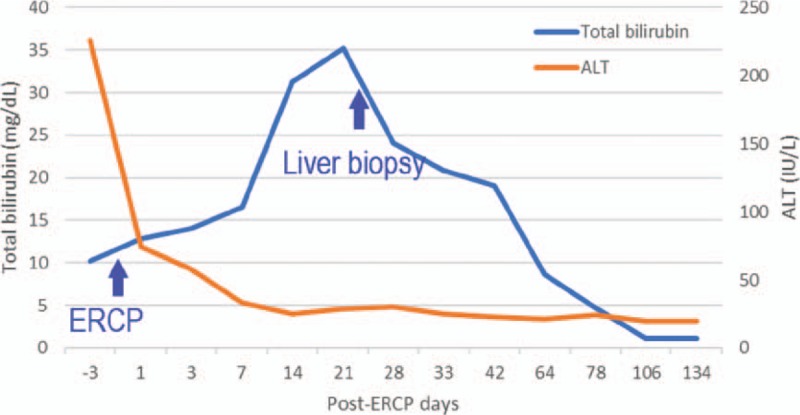
The clinical course of the patient.

**Figure 2:**
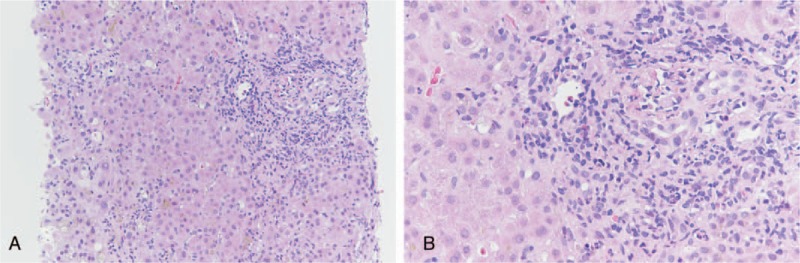
(A) Liver biopsy result showing marked cholestasis with portal and periportal inflammatory cells aggregation (H&E stain, ×200). (B) Portal inflammation consisting of lymphocytes, eosinophils, and neutrophils. No granuloma or plasma cells infiltrate are seen (H&E stain, ×400). H&E = hematoxylin and eosin.

Under the impression of obstructive jaundice, ERCP was performed after pre-treatment with intravenous cefazolin and an intramuscular injection of meperidine. A biliary tree injection of 14 cc of an ionic, high-osmolarity, iodinated contrast agent (sodium and meglumine ioxitalamate, Telebrix35, Guerbet, France) showed a filling defect in the common bile duct. Following a biliary sphincterotomy, a black stone was extracted using a balloon catheter. The patient had neither abdominal pain nor fever after the procedure. However, the serum bilirubin continued rising and was accompanied by severe pruritus. An abdominal computed tomography scan showed pneumobilia in the common bile duct, without the presence of any residual stones. Oral ursodeoxycholic acid (UDCA) (400 mg 3 times daily) and cholestyramine (4 g 3 times daily) were prescribed from 3rd day after ERCP. Total serum bilirubin increased up to 35.2 mg/dL on the 21st day after ERCP (Fig. 1). For the study of unusual prolonged cholestasis following ERCP, a percutaneous liver biopsy was performed. Histology revealed marked cholestasis with portal inflammation, consisting of lymphocytes, eosinophils, and neutrophils. No granuloma or plasma cells infiltrate were observed. Drug-related cholestasis was considered (Fig. 2). From the 7th week after ERCP, his symptoms and markers of cholestasis were improving. Serum total bilirubin returned to be normalized (1.1 mg/dL) on the 106th day after ERCP. The patient understood the disease course as a complication of contrast agent. The patient has provided informed consent for publication of the case.

## 3 Discussion

ERCP provides a valuable diagnostic tool and possible therapeutic intervention for the patients with obstructive jaundice. Using sphincterotomy or papillary balloon dilatation and subsequent stone removal, ERCP is currently the standard endoscopic therapy for the treatment of choledocholithiasis related obstructive jaundice. If hyperbilirubinemia does not resolve after ERCP, there is possibility of incomplete biliary drainage due to a retained stone, blood clot, poorly functional biliary stent, ascending cholangitis, or less frequently drug-induced cholestasis. Further imaging for differential diagnosis is mandatory.

Prolonged cholestasis is considered a rare complication of ERCP.^[3–8]^ The exact mechanism remains unclear, although it is speculated due to an allergy to the contrast agent or the premedication before ERCP.^[4]^ Liver injury due to idiosyncratic reaction was postulated. The adverse reaction may be contributed to the contrast material being infused under high pressure, which can have a toxic effect on the liver with disruption of canalicular plasma membranes.^[3]^ Systemic distribution of the contrast medium from the bile duct and the spreading of the agent extracellularly to the nearby tissues might be responsible for direct toxic liver injury.^[8]^ However, the osmolality, ionic nature, and injected volume do not seem to be associated with the risk of injury. A “re-challenge test” could confirm the diagnosis, but it was believed that this might expose the patient to potential risks and was unethical. In some reported cases, the repeated cholangiography in diagnosis for residual choledocholithiasis occurred as an unintentional, re-challenge test to lead more prolonged cholestasis.^[3,5,6,8]^ The cause-result time connection between the use of a contrast agent and increase of bilirubin indicated the possibility of toxicity and strengthened the association.^[8]^ Cefazolin and pethidine were given as a premedication for our patient. These agents have been reported to cause cholestasis.^[9,10]^ Thus, additional liver cell damage caused by these agents should not be dismissed. Only re-challenge test can exclude their effect of hepatotoxcity, but it is impractical in this ill patient. From the pathophysiology of direct injury in bile duct, we believe that the contrast is most likely offending agent of prolonged cholestasis.

We performed a systematic search of the MEDLINE database with keywords of “cholestasis” and “ERCP” in the published articles from January 1, 1970, to January 1, 2019, and there were 8 reported cases with prolonged cholestasis more than or equal to 30 days (Table 1). In these 9 patients (including our patients), the mean age was 53 ± 11 years old, and 67% of the patients were male. Three patients had ever received second ERCP due to concerns of a retained stone or inadequate biliary drainage, and the jaundice worsened following the re-challenge test using the contrast agent. The mean peak of total serum bilirubin was up to 20.4 ± 11.8 mg/dL (range: 6.8–45.3 mg/dL), and occurred on 16th ± 7th days (range: 6th–28th days). Jaundice recovered after mean time of 54th ± 22th days (range: 30th–106th days). UDCA and cholestyramine were the most used medications. Five patients responded well to prednisolone 30 to 50 mg per day. The prognosis was favorable without long-term consequences.

**Table 1:**
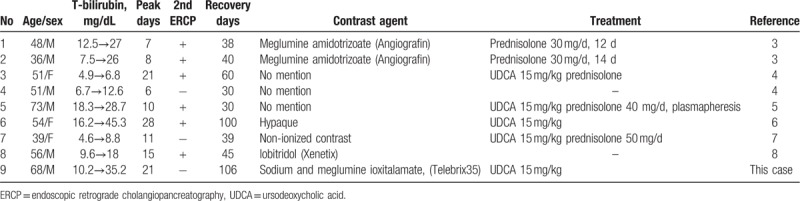
The course and treatment of 9 cases of prolonged cholestasis after endoscopic retrograde cholangiopancreatography.

There is currently no standard medical therapy for post-ERCP cholestasis. Combined UDCA at a dose of 15 mg/kg and cholestyramine at 4 g 3 times per day was successful in 1 patient.^[6]^ Prednisolone treatment at 30 mg/d had been reported in refractory UDCA cases.^[3,4]^ It has been suggested that a short course of glucocorticoids may improve biochemical abnormalities that persist following UDCA.^[4]^ Plasmapheresis had been used in patients with persistent pruritis and cholestasis despite treatment with UDCA, cholestyramine, and glucocorticoids.^[5]^

Prolonged cholestasis is a rare complication associated with ERCP. Clinicians should be aware of this uncommon side effect of the contrast agents. Using noninvasive imaging instead of repeated cholangiography to ensure the clearance of bile duct stones can avoid the worsening of jaundice again. Although the cholestasis was usually prolonged, the outcome was favorable without long-term consequences for the patient.

## Author contributions

**Conceptualization:** Cheng-Kuan Lin.

**Investigation:** Cheng-Kuan Lin, Wen-Chih Huang.

**Writing – original draft:** Cheng-Kuan Lin.

**Writing – review and editing:** Cheng-Kuan Lin, Wen-Chih Huang.

Cheng-Kuan Lin orcid: 0000-0002-0446-3606.

## References

[1] Adler DG Baron TH Davila RE American Society for Gastrointestinal Endoscopy. ASGE guideline: the role of ERCP in diseases of the biliary tract and the pancreas. Standards of Practice Committee of American Society for Gastrointestinal Endoscopy. Gastrointest Endosc 2005;62:1–8. 15990812 10.1016/j.gie.2005.04.015

[2] Anderson MA Fisher L Jain R ASGE Standards of Practice Committee. Complications of ERCP. Gastrointest Endosc 2012;75:467–73. 22341094 10.1016/j.gie.2011.07.010

[3] Dourakis SP Mayroyannis C Alexopoulou A Prolonged cholestatic jaundice after endoscopic retrograde cholangiography. Hepatogastroenterology 1997;44:677–80. 9222670

[4] Lee HM Bonis PAL Kaplan MM Persistent cholestatic jaundice after ERCP. Am J Gastroenterol 2006;101:204–5. 10.1111/j.1572-0241.2006.00393_7.x 16405561

[5] Saritas U Aydin B Ustundag Y Plasmapheresis and corticosteroid treatment for persistent jaundice after successful drainage of common bile duct stones by endoscopic retrograde cholangiography. World J Gastroenterol 2007;13:4152–3. 17696241 10.3748/wjg.v13.i30.4152 PMC4205324

[6] Patani O Foulkes SL Njie R Prolonged cholestasis induced by endoscopic retrograde cholangiopancreatography. Frontline Gastroenterol 2010;1:121–4. 28839560 10.1136/fg.2009.001099 PMC5536780

[7] Musa AA Worsening cholestasis after endoscopic retrograde cholangiography. J Med J 2012;46:65–8.

[8] Tziatzios G Gkolfakis P Papanikolaou IS An unusual case of prolonged post-endoscopic retrograde cholangiopancreatography jaundice. Hepatobiliary Pancreat Dis Int 2016;15:220–2. 27020640 10.1016/s1499-3872(15)60402-7

[9] Kluender CN Klein R Kohler B Dramatic increase in bilirubin after ERCP-pethidine as a possible cause of drug-induced hepatitis. Z Gastroenterol 2003;41:1157–60. 14661125 10.1055/s-2003-45279

[10] Ammann R Neftel K Hardmeier T Cephalosporin-induced cholestatic jaundice. Lancet 1982;320:336–7. 10.1016/s0140-6736(82)90311-7 6124751

